# Cervical cancer and COVID—an assessment of the initial effect of the pandemic and subsequent projection of impact for women in England: A cohort study

**DOI:** 10.1111/1471-0528.17098

**Published:** 2022-02-06

**Authors:** Jennifer M. Davies, Alice Spencer, Sian Macdonald, Lucy Dobson, Emily Haydock, Holly Burton, Georgios Angelopoulos, Pierre Martin‐Hirsch, Nick J. Wood, Amudha Thangavelu, Richard Hutson, Sarika Munot, Marina Flynn, Michael Smith, Bridget DeCruze, Eva Myriokefalitaki, Katelijn Sap, Brett Winter‐Roach, Robert Macdonald, Richard J. Edmondson

**Affiliations:** ^1^ Liverpool Women’s NHS Foundation Trust Liverpool UK; ^2^ Lancashire Teaching Hospitals NHS Trust Preston UK; ^3^ Leeds Teaching Hospitals NHS Trust Leeds UK; ^4^ Hull University Teaching Hospitals NHS Trust Hull UK; ^5^ Christie NHS Foundation Trust Manchester UK; ^6^ Division of Cancer Sciences, Faculty of Biology, Medicine and Health, St Mary’s Hospital University of Manchester Manchester UK; ^7^ Department of Obstetrics and Gynaecology, Manchester Academic Health Science Centre, St Mary’s Hospital Manchester University NHS Foundation Trust Manchester UK

**Keywords:** cervical cancer, chemotherapy, diagnosis, gynaecological cancer, palliative care, radiation therapy, surgery

## Abstract

**Objective:**

To review the effect of the COVID‐19 pandemic on the diagnosis of cervical cancer and model the impact on workload over the next 3 years.

**Design:**

A retrospective, control, cohort study.

**Setting:**

Six cancer centres in the North of England representing a combined population of 11.5 million.

**Methods:**

Data were collected retrospectively for all diagnoses of cervical cancer during May–October 2019 (Pre‐COVID cohort) and May–October 2020 (COVID cohort). Data were used to generate tools to forecast case numbers for the next 3 years.

**Main outcome measures:**

Histology, stage, presentation, onset of symptoms, investigation and type of treatment. Patients with recurrent disease were excluded.

**Results:**

406 patients were registered across the study periods; 233 in 2019 and 173 in 2020, representing a 25.7% (*n* = 60) reduction in absolute numbers of diagnoses. This was accounted for by a reduction in the number of low stage cases (104 in 2019 to 77 in 2020). Adding these data to the additional cases associated with a temporary cessation in screening during the pandemic allowed development of forecasts, suggesting that over the next 3 years there would be 586, 228 and 105 extra cases of local, regional and distant disease, respectively, throughout England. Projection tools suggest that increasing surgical capacity by two or three cases per month per centre would eradicate this excess by 12 months and 7 months, respectively.

**Conclusions:**

There is likely to be a significant increase in cervical cancer cases presenting over the next 3 years. Increased surgical capacity could mitigate this with little increase in morbidity or mortality.

**Tweetable Abstract:**

Covid will result in 919 extra cases of cervical cancer in England alone. Effects can be mitigated by increasing surgical capacity.

## Tweetable Abstract

Covid will result in 919 extra cases of cervical cancer in England alone. Effects can be mitigated by increasing surgical capacity.

**Linked article** This article is commented on by Leslie Stewart Massad, pp. 1140 in this issue. To view this minicommentary visit https://doi.org/10.1111/1471-0528.17100.

## 1 INTRODUCTION

Cervical cancer is a disease which is both preventable and treatable, although achieving this requires an effective screening and early detection programme with access to appropriate treatments when required.^1^ All elements of this programme have been affected by the COVID pandemic, but whereas in the UK, there has been a focus on maintaining and then quickly restoring cancer treatments, maintaining the screening programme and the diagnostic service have proved more difficult, and significant gaps have occurred.

In contrast to many cancers, the natural history of the disease is well understood and comprises a noninvasive latent phase of HPV infection and cervical intraepithelial neoplasia, during which screening and prevention can take place, followed by an early invasive phase in which disease remains localised to the cervix and treatment is usually curative.^2^ Although short delays in treatment are not associated with significant stage shift,^3^ longer delays will be associated with disease progression^4^ and increased loss of fertility with worsening of overall survival.

The disruption to normal healthcare services caused by the pandemic is likely to lead to increased numbers of cervix cancers being diagnosed during the recovery period.^5^ This may occur for one of four identified reasons; restriction of access to primary care due to staff sickness or shortages; reduction in treatment capacity following diagnosis due to limitations within the hospital service; reduction of screening and investigation services; and women either not attending for screening or not seeking help with symptoms due to concerns about the risk of contracting COVID‐19 when attending health services.^6^

These four factors will lead to ‘extra’ cases, which in turn can be divided into two discrete groups; ‘hidden’ and ‘excess’ cases. Hidden cases may be defined as existing cases of invasive cancer, which have not yet been diagnosed but will present at some point following resumption of diagnostic services. These cases would always have occurred but diagnosis has been delayed as a result of the pandemic. In contrast, excess cases may be defined as cases of invasive disease which have only occurred because of a lack of access to screening services. These cases would not have existed without the pandemic.

Estimates of the number of excess cases for England have already been calculated^7^ and suggest that there could be up to 630 excess cases of invasive disease diagnosed in England over the next 3‐year period. In contrast, with the exception of some early modelling studies which suggested possible impacts of lack of diagnostic services,^8^ there are few real world data to estimate the numbers of hidden cases of invasive disease.

We have therefore used clinical data, derived from six major cancer centres, based in the North of England, to investigate the impact of the pandemic on diagnostic rates of cervical cancer. We have then combined this with published data related to the impact of the pandemic upon screening, to forecast how this will impact workload over the next 3‐year cycle. Finally, we have projected the impact of increasing treatment capacity on this increased workload.

## 2 METHODS

### 2.1 Data collection

Registry data can be a useful source of diagnostic data, but often lack information on stage and mode of presentation.^9^ We therefore carried out a retrospective, historical control, cohort study. Data were collected from six major cancer centres covering a contiguous geographical area in the North of England and serving a population of 11.5 million people, thus representing 20.5% of the population of England.

Data from each Cancer Centre (St Mary’s and Christie’s in Manchester, Liverpool, Preston, Leeds and Hull) were collected retrospectively for two equal time periods of 6 months each, prior to and during the COVID‐19 pandemic; May–October 2019 (representing a Pre‐COVID cohort) and May–October 2020 (representing a COVID cohort.) Data were collected by senior members of the clinical teams and included all cervical cancers diagnosed during the time period. Data including histology, stage at diagnosis, mode of presentation, date of onset of symptoms (where available), investigation and type of treatment were collected. Patients with recurrent disease were excluded. All cases were converted to the FIGO 2018 staging^10^ for consistency of analysis. For patients presenting to healthcare services, the date of onset of symptoms, where available, was recorded; where no exact date was specified, the first date of the month symptoms began was recorded. Date of diagnosis was considered as date of histopathological report confirming cervical cancer and date of first attendance was considered as the date of first appointment with a specialist. If patients received more than one type of treatment (for example chemotherapy followed by radical hysterectomy) they were categorised to the most radical treatment group. All centres use an electronic database to collate cancer centre data and outcomes. Data were not available or collected regarding primary care. Hence this study only relates to patient presentation and treatment at the tertiary level cancer centres, not the impact of COVID on initial presentation, investigation or diagnosis in primary care.

### 2.2 Data handling and modelling

Data were anonymised prior to collation and all data handling took place in MS EXCEL. Shapiro–Wilks test was used to assess normality with chi‐square and *t*‐tests for parametric data and Mann–Whitney *U‐*tests for non‐parametric data. The binomial hypothesis test was used to test for difference in staging. Statistical significance was considered if *P‐*values were <0.05.

Forecasting and projection tools were developed in MS EXCEL and were limited to estimates for England (other UK jurisdictions managed COVID differently and also have different cervical screening protocols, thus prohibiting extrapolation to the devolved nations in the UK). Tools were developed to make forecasts (not including the effects of interventions) and projections (including the effects of interventions).^11^ Assumptions were taken from published sources as stated in the text.

## 3 RESULTS

### 3.1 Numbers of new cases were reduced during the pandemic

A total of 406 new patients were registered with the six participating centres across the two study periods; 233 in 2019 (pre‐COVID) and 173 in 2020 (COVID), representing a 25.7% (*n* = 60) reduction in the number of cases diagnosed between the two study periods (Table 1).

**TABLE 1:** Patient characteristics

	Pre‐COVID *n* (%)	COVID *n* (%)	Total	*P*‐value
Overall numbers	233	173	406	0.003^a^
Treating hospital				
A	63 (27)	35 (20)	98	0.06^b^
B	12 (5)	11 (6)	23	
C	68 (29)	51 (29)	119	
D	21 (9)	21 (12)	42	
E	50 (21)	43 (25)	93	
F	20 (9)	12 (7)	32	
Total	233	173		
Stage at diagnosis				
1a1	53 (23)	40 (23)	93	0.04^c^
1a2‐1b2	51 (22)	37 (21)	88	
1b3	5 (2)	2 (1)	7	
2	42 (18)	16 (9)	58	
3	31 (13)	36 (21)	67	
4	26 (11)	21 (12)	47	
Not documented	25 (11)	21 (12)	46	
Total	233	173	406	
Histology				
SCC	162 (70%)	115 (66)	277	0.7^b^
Adenocarcinoma	50 (21%)	34 (20)	84	
Neuroendocrine	1 (0.4%)	2(1)	3	
Undifferentiated	1 (0.4%)	0 (0)	1	
Small cell Ca	1 (0.4%)	0 (0)	1	
Adenosquamous	0 (0%)	8 (5)	8	
Not documented	18 (8%)	14 (8)	32	
Total	233	173	406	
Symptoms				
Pain/Bleeding	78 (33)	69 (40)	147	0.5^b^
Abnormal smear	60 (26)	43 (25)	103	
Unknown	95 (41)	61 (35)	156	
Total	233	173	406	
Mode of presentation				
GP	63 (27)	54 (31)	117	0.04^b^
Colposcopy/abnormal smear	60 (26)	49 (28)	109	
A + E	15 (6)	10 (6)	25	
Other	30 (13)	24 (14)	54	
Unknown	65 (28)	36 (21)	101	
Total	233	173		
Time of symptoms to diagnosis				
Median	21.5	19		0.7^d^
Range	1‐474	1‐668		

^a^ Chi‐square.

^b^ Paired *t*‐test.

^c^ Chi‐square comparing early stage versus late stage.

^d^ Mann–Whitney *U‐* test.

The six participating centres together, represent a contiguous geographical area in the North of England covering a population of 11.5 million people. Extrapolating these data to the rest of England (population 55.9 million) suggests that up to 273 cases of cervical cancer remained undiagnosed by the end of the study period, which represented the time of most disruption to diagnostic services.

Although not achieving significance, there was a 28% reduction in number of asymptomatic cases diagnosed through abnormal cytology compared with only an 11% reduction in symptomatic cases, *P* = 0.04 (Table 1). Time from symptom onset to presentation was similar in both time periods (Pre‐COVID: median 21.5, range 1–474 days, and COVID: median 19, range 1–668 days) (*P* = 0.7 Mann–Whitney *U*‐test).

### 3.2 Analysis by FIGO Stage suggests that ‘hidden’ cases are low stage and therefore potentially curable

Mode of treatment, and subsequent outcome, is almost entirely driven by stage of disease at presentation. Disease presenting at FIGO stage 1B2 or below is usually treated surgically with high survival rates and for some cases the option of fertility preservation. FIGO stage 1B3/2/3 is treated with concurrent chemoradiotherapy with curative intent but loss of fertility. Stage 4 disease is treated with systemic therapies with or without radiotherapy with palliative intent.

Dividing the cohort into these three groups by stage (Table 2) confirms that the majority of ‘hidden’ cases are likely to be stage1B2 or below and would be treated by surgery alone with curative intent if they remain at this stage by the time of presentation.

**TABLE 2:** Confusion matrix showing cases categorised by stage

	Pre‐COVID (*n*)	COVID (*n*)
≤1B2 (surgery)	104	77
1B2/2/3 (curative RT)	78	73
4 (palliative RT/chemo)	26	21

### 3.3 Forecasting the presentation pattern of extra cases over a 3‐year period

**FIGURE 1:**
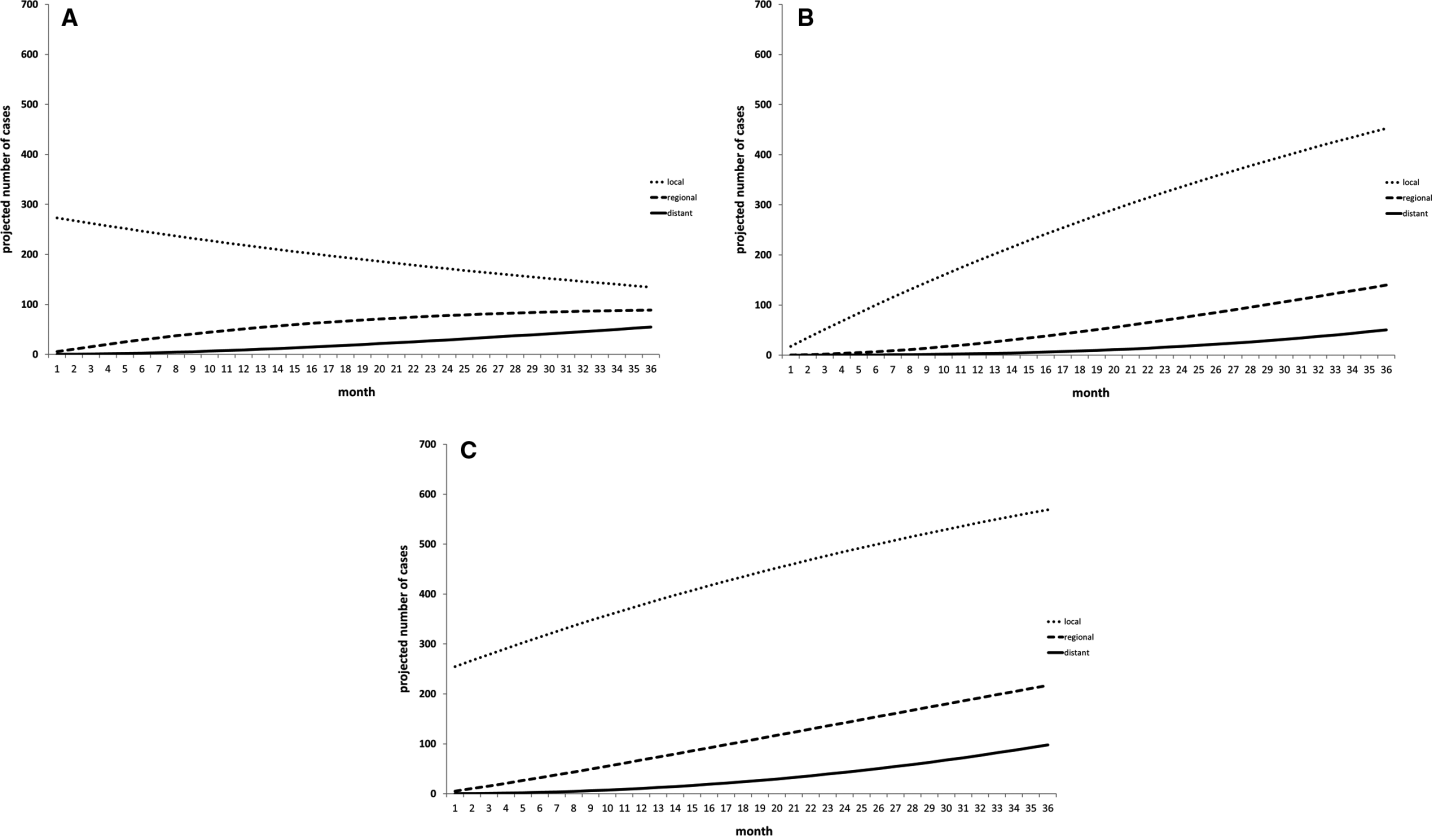
Forecasts of likely impact of COVID on extra cases of cervical cancer with no increased treatment capacity. Number of cases for each forecast represents numbers expected in addition to normal background diagnostic rates. Forecasts have been generated for a 3‐year period to reflect the normal cycle of cervical screening. (A) Model of ‘hidden’ cases showing effect of 273 additional cases. (B) Model of ‘excess’ cases showing effect of 630 additional cases presenting over a 3‐year period. (C) Summation of (A) and (B) to show likely increase in cases over three periods if no additional treatment capacity is provided

By definition, the hidden cases already exist as invasive cancers. Although it appears most of these are early stage, timely diagnosis and treatment are required to prevent progression to more advanced disease. To visualise this, we assumed progression rates of 0.020 and 0.025 per month for progression from local to regional, and regional to distant, respectively^4^ to forecast the likely progression of the 273 hidden cases in England over a 3‐year period, assuming that none receive treatment (Figure 1A). With no additional treatment capacity available, this would equate to a progression to 88 additional cases of regional disease and 54 cases of distant disease by 3 years.

In addition to the hidden cases, the cervical screening programme in England was paused completely for a 3‐month period between April and June 2020 and only partially restored after that leading to a delay in screening of approximately 6 months.^7^, ^12^ It has been estimated that this pause will result in an excess of 630 cases over a 3‐year period.^7^ In contrast to the hidden cases, these are likely to present at a steady rate over the 3‐year period, as modelled in Figure 1B.

Summation of both hidden and excess cases suggests that at the end of the 3‐year period extra cases throughout England, over and above normal workload, would be 586, 228 and 105 cases for local, regional and distant disease, respectively (Figure 1C).

### 3.4 Projecting the effects of increased treatment capacity

**FIGURE 2:**
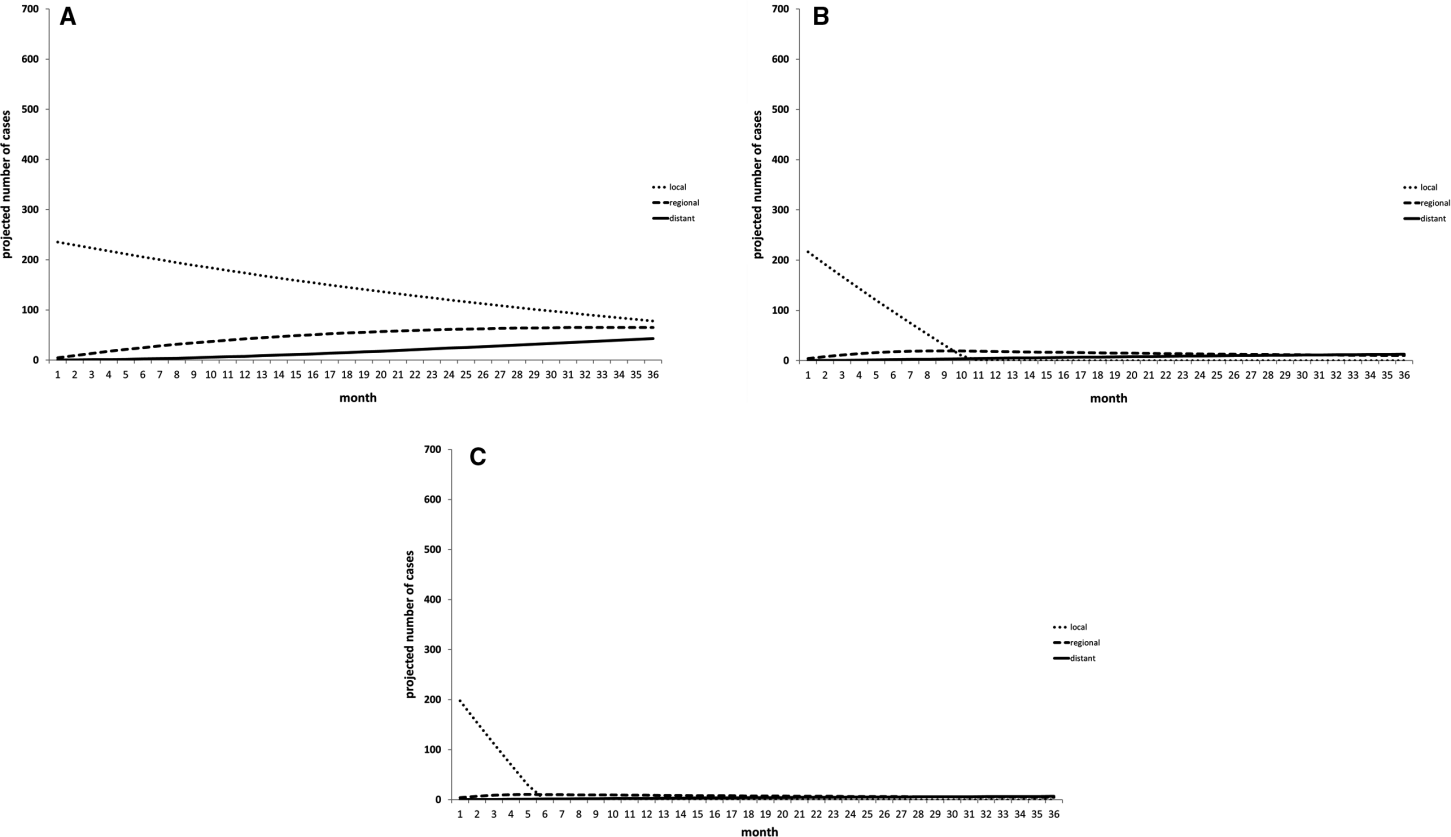
Projections of the effect of increasing treatment capacity on increased numbers of cervical cancer cases presenting as result of COVID pandemic. The projection generated in Figure 1C was used as a baseline representing the additional cases expected as a result of the pandemic. The effects of increasing by one, two or three cases per month per cancer alliance in England are estimated in (A), (B) and (C), respectively, showing that increasing capacity by three cases per month per cancer alliance, would eradicate the extra low stage disease within 7 months with minimal impact on rates of regional and distant disease

Finally, we developed a projection tool to study the effects of increasing treatment capacity. Given that the majority of extra cases will be at early stage at presentation, we studied the effect of additional surgical capacity to treat these extra cases. Cancer services in England are managed through 19 ‘Cancer Alliances’. We studied the effect of increasing surgical capacity by one, two or three extra cases per cancer alliance per month (Figure 2A, B and C, respectively).

Increasing capacity by one case per alliance per month would enable treatment of some of the extra cases, leaving 221 remaining excess cases at the end of the 3‐year cycle, with 76 and 50 being regional and distant disease, respectively. In contrast, increasing the capacity by two or three cases per month would eradicate the local disease group entirely by 12 months and 7 months, respectively, but leave small residual numbers of regional and distant disease (29 regional and distant cases when capacity increased by two per month (Figure 2B) and 14 regional and distant cases when capacity increased by three per month (Figure 2C)).

## 4 DISCUSSION

### 4.1 Main findings and interpretation

Here we have reported data from a retrospective historical control series showing, for the first time, the actual effects of the pandemic on diagnostic rates of cervical cancer in a large contiguous population. These data support much of the modelling, prediction and speculation that have suggested that cancer cases are likely to have gone undetected during the pandemic. Furthermore, we have been able to identify that the undiagnosed cases are likely to be predominantly low stage, a point in the progression of the disease which is usually curable if treated promptly.

Specifically, we have identified that the rate of cervical cancer diagnosis was down by 25% during the period of the first wave of COVID infection in England. Although cervical cancer is a relatively rare cancer, this still equates to over 230 cases which have not been diagnosed in a timely manner. Failure to do so will have long‐term impacts if these cases are not rapidly identified and treated.

Taken with predictions of an excess of cases caused by a 6‐month pause in cervical screening,^7^ the total number of extra cases of cervical cancer that are likely to present in England over the next 3 years is over 860. However, our projections would suggest that if cases can be diagnosed quickly, and treatment capacity can be increased, women could receive therapy before their disease progresses to a stage requiring radical radiotherapy or palliative systemic anti‐cancer treatment. We would therefore argue that resources should be focused on increasing diagnostic capacity and access to surgical treatments, which form the mainstay for treatment of early stage disease.

In reality, increasing surgical capacity is likely to be a challenge. The number of surgeons with the ability to carry out radical surgery for cervical cancer is limited and all surgical oncology services are faced with pressure from increasing numbers of cancers of all types, meaning that even getting access to operating theatre time can be challenging. Furthermore, most gynaecological oncology services were working at or above capacity before the pandemic and so a demand to increase capacity further may prove to be impossible.

### 4.2 Strengths and limitations

This study utilised clinical data collected by senior clinicians directly from clinical and MDT records, giving confidence to the veracity of the data. Although registry data can provide information covering large geographical areas, important data items such as stage at presentation are often missing,^9^ limiting the utility of such data collections.

In this study we have been able to accrue data related to a population of 11.5 million people, representing over 20% of the population of England, suggesting that the findings are likely to be a fair representation of the picture in England. The devolved nations and other jurisdictions may have different findings given their different approach to management of the pandemic and, in particular, variable access to hospital services during this time.

The use of a historical control does not exclude the possibility of another confounder to explain the apparent reduction in incidence over these two time periods. An interrupted time series design for the study, with repeated observations pre‐ and post‐pandemic, would have allowed for correction of other confounders, but data from such a study will not be available for some years to come, during which cases will continue to accrue. The presence of other confounders in fact seems unlikely; although there has been a decrease in incidence of cervical cancer over the last two decades, the incidence has plateaued over the last 5 years up to and including 2019.^13^

By choosing a period of time from May to October 2020, we have included the time of most upheaval to the NHS. It is probable that further extra cases may accrue as a result of delays in the recovery programme and further audits will be required to monitor this and update the modelling in due course. The forecasts presented here represent the best estimates for the likely increased workload over the next 3 years.

## 5 CONCLUSION

We have demonstrated that there will be a significant uplift in numbers of cases of cervical cancer presenting over the next 3 years as a result of both lack of diagnosis of established cases and an excess of cases caused by lack of screening. We have also demonstrated that increases in surgical capacity, while a challenge to achieve, would be capable of mitigating this increase with little increase in morbidity or mortality.

## DISCLOSURE OF INTERESTs

None declared. Completed disclosure of interest form are available to view online as supporting information.

## CONTRIBUTIONS TO AUTHORSHIP

JD, AS, RM, RE: data collection, analysis, writing of the paper. SM: statistics. LD: review of the paper, references. EH, HB, GA, PM‐H, NW, AT, RH, SM, MF, MS, BD, EM, KS, BW‐R: data collection, review of the paper. RM: data collection, analysis, Writing of the paper.

## DETAILS OF PATIENT CONSENT

Patient’s (or next of kin’s) permission for publication was not required.

## DETAILS OF ETHICS APPROVAL

No ethical approval was required for this study.

## Supporting information


Data S1

Data S2

Data S3

Data S4

Data S5

Data S6

Data S7

Data S8

Data S9

Data S10

Data S11

Data S12

Data S13

Data S14

Data S15

Data S16

Data S17

Data S18

Data S19

Data S20

## Data Availability

The data that support the findings of this study are available on request from the corresponding author. The data are not publicly available due to privacy or ethical restrictions.
